# Molecular Mechanisms and Emerging Precision Therapeutics in the Gut Microbiota-Cardiovascular Axis

**DOI:** 10.7759/cureus.83022

**Published:** 2025-04-26

**Authors:** Jhon Alexander Ponce Alencastro, Diego Alejandro Salinas Lucero, Ricardo Perez Solis, Carlota Griselda Herrera Giron, Andrés Sebastián Estrella López, Pablo Xavier Anda Suárez

**Affiliations:** 1 Geriatrics, Universidad Técnica de Manabí, Portoviejo, ECU; 2 General Practice, Virrey Solís I.P.S., Ibagué, COL; 3 Material Sciences, Instituto Tecnológico Superior de Atlixco, Tecnológico Nacional de México (TecNM), Atlixco, MEX; 4 General Practice, Facultad Mexicana de Medicina, Universidad La Salle, Mexico City, MEX; 5 Public Health, NeoScientia Consulting Group, Quito, ECU; 6 General Practice, Universidad de las Américas, Quito, ECU

**Keywords:** cardiovascular disease, gut-cvd axis, gut microbiota, molecular mechanism, precision therapeutics

## Abstract

A microbiome in the gut plays a significant role in cardiovascular health and disease. Dysbiosis is an imbalance in the gut microbiome, leading to multiple cardiovascular diseases (CVD) such as atherosclerosis, hypertension, and heart failure. Gut microbe-derived metabolites such as trimethylamine-N-oxide (TMAO) and short-chain fatty acids (SCFAs) are important mediators of the gut-heart axis. Evaluation of the relationship between the gut microbiome and host biomarkers with CVD requires the integration of metagenomics and metabolomics with meta-omics approaches. The literature review found that microbes and metabolic signatures are associated with the risk and progression of CVD. The development of precision therapeutic approaches for targeting gut microbiota includes preventing adverse microbial effects using probiotics, prebiotics, and the drug-as-bug approach to inhibit harmful metabolites of microbiomes, and fecal microbiota transplantation (FMT). However, the implication and practice of these findings in clinical settings face challenges due to the heterogeneity of study designs, difficulty in the determination of causality, and the impact of confounding factors such as diet, medication, and potential inter-individual gut microbiome variability. Future researchers are recommended to conduct longitudinal studies to further establish both gut microbiome associations with CVD and develop successful precision therapeutics approaches based on the microbiome for the treatment of CVD.

## Introduction and background

Cardiovascular disease (CVD) includes conditions such as coronary artery disease (CAD), heart failure (HF), stroke, and hypertension [1]. Worldwide, CVD remains the leading cause of death. A study on the global burden of disease reported in 2023 that CVD claimed 18.56 million lives in 2019, compared to 31.31 million lives in 1990, representing a global increase of 53.81% [2]. The increasing prevalence of risk factors, including unhealthy eating habits, lack of exercise, tobacco use, and obesity problems, has led to an increase in CVD cases, particularly in developing countries. The global impact of CVD is expected to increase in the future as aging populations and lifestyle-related diseases become more prevalent, making CVD one of the major health challenges worldwide [3].

The gut-heart axis affects cardiovascular outcomes, challenging the perception of the gut as an isolated organ. The host's immunological and metabolic balance depends on gut health, specifically intestinal barrier integrity and gut microbiota homeostasis, in this dynamic, bidirectional network. Due to these discoveries, the gut-heart axis is now considered a key component of cardiovascular health, linking nutrition, activity, age, and sex hormones to myocardial infarction (MI) outcomes. This comprehensive analysis examines gut-heart axis molecular mediators and signaling mechanisms. By studying how gut barrier integrity, microbiota composition, and systemic inflammation affect CVD, researchers are highlighting emerging therapeutic methods such as dietary modifications, probiotics, and prebiotics, as well as precision medicine, that help to reduce CVD risk and improve recovery. These findings help us comprehend the gut-heart axis as a viable cardiovascular therapy target [4].

The discovery of critical molecular processes in biology led experts to recognize the gut microbiota as a significant factor in the progression and development of CVD [4]. Research shows that the vast community of microorganisms in the human digestive tract affects cardiovascular health by altering vascular function, lipid metabolism, and inflammatory responses [5]. Research indicates that dysbiosis - an imbalance in gut microbiota - contributes to the development of hypertension, atherosclerosis, and other CVDs [6]. During metabolism, microbiota in the gut produce short-chain fatty acids (SCFAs) and trimethylamine-N-oxide (TMAO), both of which affect systemic inflammation and cardiovascular risks. Literature emphasizes the importance of understanding the molecular association between gut microbiota and CVD, as such knowledge paves the way for innovative microbiota-based therapeutic strategies [7].

The management of CVD heavily depends on primary care interventions, as they address key risk factors such as hypertension, diabetes, and lifestyle-related health problems. Primary care physicians normally offer preventive practices, timely diagnostic examinations, and standard medical treatments through lifestyle modifications, medication prescriptions, and general risk prevention strategies [8]. While primary care approaches are essential to mitigate the risk factors, primary healthcare is often lacking in addressing the complex molecular mechanisms underlying CVD. The growing and expanding recognition of gut microbiota, such as *Enterobacteriaceae* and *Streptococcus spp.*, influence on CVD requires healthcare systems to shift away from primary care models that primarily focus on generalized treatment regimes. Primary care tends to overlook individual microbiome genetic composition, profiles, and molecular response to treatments, vital for developing more precise and effective treatment approaches [9].

The primary care approach is unable to address the molecular and personalized aspects of CVD patients. The standard treatment approaches work with a single method for everyone, but these methods might fail to help different individuals who possess distinct microbiome genetic composition profiles [10]. State-of-the-art precision-based strategies are needed to devise treatments to adapt to specific biological characteristics (microbiome genetic profile) of each individual. The implementation of precision therapeutic strategies, such as microbiome-based therapies, helps healthcare professionals to devise personalized treatment protocols to target molecular levels of CVD. The utilization of precision medicine approaches enables doctors to build treatments that directly target CVD pathophysiology and improve patient clinical outcomes, lowering the CVD global impact. These are advanced methods to treat CVD to improve the health of individuals who are at risk of CVD [11].

The scope of this literature review is a comprehensive review of the gut microbiome’s composition, function, and key metabolites, particularly regarding their effects on cardiovascular health. The review investigates the molecular processes of gut microbiota to cause CVD, utilizing two separate mechanisms known as systemic inflammation and immune regulation. This review analyzes both genetic and epigenetic aspects that affect host-microbiota relationships because precision medicine requires molecular insights. Moreover, the review focused on the advancement of precision medicine, including the integration of multi-omics (genomics, metabolomics, and profiling of microbiome), personalized dietary plans, and individual microbiome-based pharmacological intervention. The review explored innovative therapeutic strategies other than conventional primary care interventions, such as next-generation prebiotics, probiotics, and fecal microbiota transplantation (FMT). It also explored adjunct therapeutic approaches that can modulate the microbiota of the gut to improve the cardiovascular health of an individual.

In the end, the review highlighted the gap between conventional primary care interventions and precision strategies to treat microbiome-mediated CVD. It discussed personalized microbial therapies, challenges, and limitations of precision therapeutics, and proposed a way forward on the basis of contemporary literature to implicate microbiome modulation in clinical practice.

## Review

Molecular mechanism associated with gut microbiota and CVD axis

Metabolic Pathways and Microbial Metabolites

The gut microbiome regulates metabolic pathways that affect cardiovascular health. Recent studies have found that microbial metabolites, including TMAO and SCFAs, affect cardiovascular health via molecular pathways [12].

Role of TMAO in atherosclerosis: TMAO is a gut microbiota-derived metabolite produced from dietary choline, carnitine, and phosphatidylcholine through microbial fermentation. Once generated, TMAO is absorbed and metabolized in the liver by flavin monooxygenases. TMAO has been linked to atherosclerosis, where it promotes the formation of foam cells in arterial plaques by enhancing macrophage uptake of oxidized low-density lipoprotein (LDL). Studies have shown that higher levels of TMAO are associated with increased risk for cardiovascular events such as heart attacks and strokes [13,14]. Moreover, TMAO inhibits bile acid (BA) synthesis, leading to reduced cholesterol excretion and contributing to cholesterol buildup in the arteries, thus worsening atherosclerosis [15]. TMAO activates nuclear factor kappa B (NF-κB) and mitogen-activated protein kinase (MAPK) signaling to exacerbate vascular damage by attracting inflammatory cells to the endothelium layer. Thus, addressing the TMAO synthesis route may reduce atherosclerosis' negative consequences [16].

Influence of SCFAs on inflammation and lipid metabolism: Fermentation of dietary fibers by gut bacteria produces three metabolites of SCFAs known as acetate, propionate, and butyrate. They are absorbed into the bloodstream to manifest various cardiovascular effects. Multiple cardiovascular effects result from metabolites that are absorbed into the bloodstream. Butyrate serves as a specific compound that enhances how well gut barriers function, according to research findings. A randomized controlled experiment by Chen et al. (2020) showed that SCFAs reduce inflammation and prevent atherosclerosis. SCFAs affect lipid metabolism via regulating cholesterol and triglyceride metabolism genes. Butyrate treatment according to Chen et al. (2020) produces elevated high-density lipoprotein (HDL) levels and reduces LDL concentrations, which helps prevent artery plaque development. Through SCFA activation of T-cells and macrophages, the body experiences decreased systemic inflammation together with better endothelial function. Lipid metabolism shows changes because SCFAs regulate genes that control cholesterol homeostasis and triglyceride metabolism [17]. Xu and Yang (2021) also demonstrated that butyrate and other SCFAs possess anti-inflammatory properties, which help prevent lipid accumulation in blood vessels [18]. Kallapura et al. (2024) also reported that microbiome-based dietary interventions improved metabolic parameters, including blood glucose and lipids, which are key factors in CVD management. The increased diversity of beneficial gut bacteria was linked to improved lipid metabolism and reduced inflammation [13].

Other bioactive metabolites and their molecular targets: The development of CVD is associated with microbial metabolites, including TMAO and SCFAs, as well as indoxyl sulfate and p-cresyl sulfate. These metabolites are derived from tryptophan and phenylalanine, respectively, and are linked to endothelial dysfunction and increased oxidative stress [14]. Tian et al. (2021) also demonstrated that indoxyl sulfate causes oxidative damage to endothelial cells, which leads to atherosclerosis development and thrombosis. The breakdown products of pomegranate polyphenols, known as urolithins, display both cardiovascular-protective effects that improve blood vessel health and decrease blood clot formation [19].

The microbe-generated metabolites, including BAs and lipopolysaccharides (LPS), significantly affect cardiovascular health. A change in BA metabolism has demonstrated an impact on lipid metabolism along with atherosclerosis progression. The research by Cao et al. (2024) demonstrated that hypertensive left ventricular hypertrophy (LVH) with microbial dysbiosis leads to changes in BA and lipid metabolism pathways, which could potentially serve as biomarkers for CVDs. The cardiovascular health effects of indole and phenylalanine metabolites were discovered to regulate inflammation and endothelial cell function [20].

The cardiovascular health of individuals depends primarily on microbial metabolites, including TMAO, SCFAs, and other bioactive compounds (Table 1). Understanding of CVD molecular mechanisms may become possible through large multicenter longitudinal studies evaluating lipid metabolism at the same time as inflammation and endothelial function, because these investigations enable the development of precision cardiovascular medicine. The progressive understanding of metabolite functions gives rise to opportunities for precision microbial treatment development for CVD.

**Table 1:** Molecular mechanism of metabolites. TMAO: Trimethylamine-N-oxide; SCFAs: Short-chain fatty acids; LPS: Lipopolysaccharide; FXR: Farnesoid X receptor; LXR: Liver X receptor; TLR4: Toll-like receptor 4; LDL: Low-density lipoprotein; TNF-α: Tumor necrosis factor-alpha; IL-6: Interleukin-6

Metabolite	Molecular Mechanism	Molecular Targets
TMAO [13,14,16]	Gut microbiota transforms choline and carnitine into TMAO. The substance promotes foam cell development while enhancing cholesterol accumulation and activating inflammatory responses within vascular endothelial cells.	Endothelial cells, macrophages, liver cells, LDL receptors, foam cells
SCFAs (Butyrate, Propionate, Acetate) [13,17,18]	Butyrate, together with other SCFAs, performs several functions in the body, including decreasing systemic inflammation and strengthening the gut barrier while controlling immune system response and lipid metabolism through gene expression modifications.	Colonocytes, endothelial cells, T-cells, macrophages
Bile Acids [20]	Bile acids influence lipid metabolism by regulating cholesterol conversion and absorption. The receptors FXR and LXR serve as targets for these compounds to control lipid and glucose metabolic processes.	Liver cells, intestinal cells, cholesterol metabolism enzymes, FXR, LXR
LPS [20]	LPS from gut bacteria activates the immune system, triggering inflammation through TLR4 signaling, leading to vascular dysfunction, increased platelet aggregation, and atherosclerosis.	Endothelial cells, macrophages, TLR4, inflammatory cytokines (TNF-α, IL-6)
Indole [19,20]	Indole, produced by tryptophan metabolism, influences the gut-brain axis, modulates immune responses, and may contribute to the regulation of endothelial function.	Endothelial cells, immune cells, gut-brain axis, tryptophan metabolism
Phenylalanine [19,20]	Phenylalanine metabolism affects inflammatory pathways and neurotransmitter synthesis. Alterations in its metabolism may contribute to endothelial dysfunction and atherosclerosis.	Endothelial cells, inflammatory pathways, lipid metabolism enzymes

Inflammatory and Immune Pathways

Contribution of gut dysbiosis to systemic inflammation: Gut dysbiosis describes an abnormal state of gut microbiota that produces substantial systemic inflammation. The condition results in proinflammatory molecule production, including LPS, which moves from the gut to the bloodstream. The innate immune response is activated through Toll-like receptor 4 (TLR4) when LPS levels increase, thus promoting vascular inflammation and atherosclerosis by elevating inflammatory cytokines interleukin-6 (IL-6) and IL-8. The IL-23/IL-22 axis controls atherosclerosis-related inflammation through its ability to preserve intestinal barrier health and stop additional dysbiosis development. Butyrate, an SCFA, alongside others in this category, functions to preserve intestinal barrier function, which hinders LPS from entering circulation and decreases overall body inflammation [21].

Immune modulation through gut microbial-derived signals: The immune system regulation by SCFAs, especially butyrate, occurs through G-protein-coupled receptors (GPR41, GPR43) activation on immune cells, which leads to leukocyte recruitment and T lymphocyte activation. Transitions between microbial signals and immune modulation occur through TMAO along with other microbial products. The gut bacteria transform dietary choline into TMAO, which activates macrophages while driving foam cell development that leads to atherosclerosis formation. The outer membrane component LPS from Gram-negative bacteria triggers inflammation and modifies endothelial cell function, which advances atherosclerosis. Therefore, microbiomes activated vascular inflammation, lipid metabolism, and immune responses in CVD pathogenesis [22].

Genetic and Epigenetic Influences

The complex interplay between genetic, epigenetic, and gut microbiome factors on CVD is crucial. Multiple researchers investigate the interplay among the aforementioned factors, especially for hypertension and atherosclerosis conditions, to explain the intricate mechanisms by which gut microbiota affects cardiovascular health.

Interactions between the composition of the gut microbiome and host genetics: Genetics determines the structure of the gut microbiota, affecting the cardiovascular system. Genomic studies conducted by Louca et al. (2020) and Zhu et al. (2022) demonstrate that genes are responsible for the genetic make-up of gut-residing microbiomes. Genetic variations of sodium transporters alongside lipid metabolism genes control the gut microbiota, its blood pressure regulatory functions, and lipid metabolic activities leading to CVD. Genome-wide association studies (GWAS) analysis uncovered various single-nucleotide polymorphisms (SNPs), which proved that genetics controls how gut microbiota processes fats as well as their vascular effects [23-26]. The interactions between host genetics, the microbiome, and epigenetic modifications play a critical role in the development of CVDs. Emerging evidence points to the gut microbiome not only as a modifier of genetic predispositions but also as a key player in epigenetic regulation through microbial metabolites such as TMAO and SCFAs. It is crucial to understand these interactions or interplay to comprehend personalized medicine approaches to incorporate genetic, microbiota, and epigenetics to predict and treat CVD (Table 2).

**Table 2:** Genomic and microbial influence on CVD, including mechanism of interaction and epigenetic modulation. CVD: Cardiovascular disease; TMAO: Trimethylamine-N-oxide; SCFAs: Short-chain fatty acids

Key Studies	Study Design and Purpose	Objective	Findings
Louca et al. (2020), [23], Zhu et al. (2022) [24]	Genomic study, GWAS (genome-wide association studies), cohort studies.	Interaction between host genetics and microbial composition	Genetic variations influence gut microbiome composition, which in turn affects lipid metabolism and blood pressure regulation. Genetic mutations in sodium transporters and lipid metabolism genes alter microbial communities, impacting CVD risks like hypertension and atherosclerosis.
Nayor et al. (2021) [25]	Clinical and experimental study on microbial metabolites, epigenetics, and inflammatory pathways.	Epigenetic modifications driven by microbial metabolites	Microbial metabolites like TMAO and SCFAs induce epigenetic changes by altering DNA methylation and histone modifications. TMAO promotes endothelial dysfunction and foam cell formation, while SCFAs like butyrate inhibit histone deacetylases (HDACs), reducing inflammation and improving endothelial function.

Precision medicine approaches in cardiovascular therapy

Integrative Multi-omics Analysis

Integrative multi-omics analysis combined the use of genomics, metabolomics, and microbiome profiling. It promises to identify patient-related risk factors, specifically CVD. The observation and examination of the interplay of these factors across different biological levels is crucial for understanding mechanisms and individual susceptibility to CVD.

The synergy of multi-omics: Integrating metagenomics with metabolomics helps identify microbial strains and understand their roles. It clarifies the gut microbiome's significance in CVD. Combining omics methods illuminates gene regulation and links bacteria to metabolites [27]. For example, integrating metagenomics (analyzing microbial genes) with metabolomics (analyzing small molecule metabolites) can help researchers understand how changes in the gut microbiome (genomic information) translate into altered metabolic outputs that might affect host physiology [27].

CVD risk: Several sources highlight the application of integrated omics to study CVD risk. For instance, a study characterized the gut microbiome and metabolome in individuals with varying cardiovascular risk based on the Framingham score, identifying species and metabolic pathways associated with increased risk [28]. Another paper addresses how meta-omics technology can uncover the complex relationships between exercise, the microbiome, the metabolome, and cardiometabolic health, which affects atherosclerosis [29].

Fecal metabolome's importance: Research suggests that the fecal metabolome exhibits the strongest association with the gut microbiome compared to urine and plasma metabolomes. This implies that analyzing fecal metabolites in conjunction with microbiome profiling can be particularly informative for understanding gut-related influences on health risks. A substantial proportion of microbial diversity can be explained by fecal metabolites, and fecal metabolomes have a higher number of differentially expressed metabolites associated with disease [30].

Identifying specific biomarkers: Integrative analyses are used to identify potential biomarkers for disease risk and progression. For example, one study integrated metagenomics and metabolomics in CAD patients and healthy controls to identify specific microbial and metabolite features associated with CAD severity [31]. Another study used multi-omics analyses of fecal and serum samples from individuals with stable CAD and type 2 diabetes to link microbiome features to disease severity and identify prognostic bacterial biomarkers [19].

The gut-heart axis: The interplay between the gut microbiome and dietary-derived metabolites significantly affects the pathogenesis of CVDs, a concept known as the gut-heart axis [32]. Meta-omics approaches help to unravel host-microbiome interactions and assess causality in clinical settings. Researchers can identify microbial metabolites such as TMAO and SCFAs, which are implicated in atherosclerosis [18].

TMAO Levels: Discovery and Validation

Biomarker discovery and validation are implications of integrative multi-omics analysis. It helps to examine measurable physiological or pathological indicators. The potential biomarkers are TMAO levels and SCFA profiles to discover and validate in CVD. TMAO has been identified as a metabolite linked to the gut microbiome and dietary factors like carnitine and choline. Gut microbes produce trimethylamine (TMA) from these dietary components, which is then oxidized to TMAO in the liver. Metabolomics studies have frequently found TMAO as a major source of variation in various physiological and pathophysiological states [33].

Association with CVD: Increased TMAO levels have been linked to an increased risk of CVD, such as atherosclerosis, acute coronary syndrome (ACS), carotid atherosclerosis, HF, MI, stroke, and all-cause mortality [34]. Literature has shown that TMAO can be an independent risk factor for the development of atherosclerosis and a strong predictor of future cardiovascular events [35].

Potential mechanisms: TMAO has been implicated in promoting atherosclerosis and thrombosis due to its pro-inflammatory and pro-thrombotic properties. It may also augment the production of pro-inflammatory molecules, contribute to fat accumulation in foam cells, alter reverse cholesterol transport, and increase inflammatory markers [35]. In animal studies, TMAO precursors cause foam cell development and atherosclerosis, suggesting a microbiota-dependent mechanism [6].

Validation: The association between TMAO and CVD has been validated in multiple large cohort studies over extended periods, even after adjusting for traditional risk factors. TMAO levels can predict outcomes in patients with HF and stable coronary atherosclerosis [6]. However, some research questions whether the correlation is causative or if TMAO is just a surrogate marker. Factors like diet, gut microbial flora, drug administration, and liver function can influence TMAO levels, which need consideration in its use as a biomarker [34]. Future research should identify CVD molecular pathways and investigate TMAO and its precursors as therapeutic targets.

SCFAs: Discovery and Validation

SCFAs like butyrate, propionate, and acetate are produced by the gut microbiome through the fermentation of dietary fibers. They are key metabolites that can influence host cardiovascular health.

Association with CVD: Altered levels of SCFAs have been observed in various conditions. For example, stroke patients have shown low fecal SCFA levels. Patients with ACS had significantly lower plasma propionate and butyrate levels compared to healthy controls. HF/CAD dysbiosis is linked to low microbial butyrate production [36].

Potential mechanisms: SCFAs, particularly butyrate, have been proposed to have beneficial effects by decreasing inflammation, serving as an energy source for colonocytes, influencing glucose homeostasis, and potentially reducing cholesterol levels [36].

Validation: While fecal SCFAs may provide a more direct measure of microbial activity, their circulating levels can be low, potentially limiting their use as soluble biomarkers. Studies are needed to confirm the atheroprotective effects of SCFAs and whether supplementation has long-term benefits. The causal relationships between the gut microbiome, SCFAs, and metabolic diseases are being investigated [37].

Integrative Multi-omics for Enhanced Biomarker Discovery and Validation

Combining metabolomics and microbiomics: Several sources emphasize the power of integrating metabolomics and microbiome analysis for biomarker discovery. By analyzing both microbial composition/function and their metabolic outputs, researchers can gain a more holistic understanding of disease-associated changes [38]. For instance, changes in gut microbiota composition can lead to altered levels of metabolites like TMAO and SCFAs, which in turn can influence CVD pathogenesis.

Disease classification and prediction: Integrated multi-omics data can be used to construct disease classifiers and predict disease progression [39]. For example, models incorporating both microbial taxa and metabolite levels have shown improved accuracy in distinguishing CAD patients from healthy controls and even different stages of CAD. Similarly, in Takayasu arteritis (TAK), a combined model of bacterial species and metabolic/lipidomic profiles showed good diagnostic accuracy [40].

Identifying functional signatures: Integrative analysis helps reveal functional signatures of diseases by linking specific microbes to altered metabolic pathways. For instance, in CAD, certain bacteria might affect atherosclerosis by modulating host metabolic pathways like taurine and sphingolipid metabolism. In LVH, associations between gut-specific microbes and metabolites of plasma had been identified, indicating pathways involved in the disease pathogenesis [41].

Personalized Dietary Interventions

Personalized dietary and pharmacological interventions are tailored on the basis of microbiome profiles in an individual. This area is of significant interest in leveraging to understand the gut microbiome to increase health outcomes. The sources provide several insights into this approach, including supporting evidence from clinical trials and discussions of potential mechanisms.

Personalized dietary interventions based on microbiome profiles: The idea is that since individuals have unique gut microbiome compositions, their responses to the same dietary interventions can vary significantly. Therefore, tailoring dietary recommendations based on an individual's specific microbiome profile could lead to more effective outcomes in managing various health conditions. The evidence from recent clinical trials is discussed here. In a randomized controlled trial of Kallapura et al. (2024), the impact of a microbiome-based targeted personalized diet (using the BugSpeaks® test) on individuals with type 2 diabetes and hyperlipidemia was evaluated. The test arm, receiving personalized nutritional recommendations based on their microbiota profiles, showed statistically significant improvements in HbA1c levels, systolic blood pressure, and C-reactive protein (CRP) levels compared to the control arm receiving standard diabetic nutritional guidance. Every participant in the personalized diet group experienced lower HbA1c levels. Gut microbiome profiling can produce better results than standardized meal recommendations for diabetic patients who wish to manage their glycemic parameters. The BugSpeaks® system operates through a program that analyzes microbial levels in user profiles to generate meals that promote healthy microorganisms and minimize dysbiosis development. The proposed method strives to restore the proper balance of the gut microbiota network, which might lower continuous inflammation and promote better metabolic well-being [13]. Madhogaria et al. (2022) also found that the gut microbiome affects the variability of drug responsiveness. It implies that personalized dietary interventions might potentially optimize the efficacy of other therapeutic approaches [42]. In their Mayo Clinic Center for customized medicine research, Xu and Yang (2021) found that a microbiome-based diet, together with genetics, age, and exercise level, controlled blood glucose better than decreasing carbs and calories. This supports the hypothesis that gut microbiota consideration might improve dietary advice [18].

Personalized diet plan mechanism of action: Personalized diets aim to modulate the gut microbiota composition and function. This can lead to changes in the abundance of specific bacteria. For instance, Kallapura et al. (2024) observed increases in *Bifidobacterium angulatum* and *Lactobacillus brevis*, bacteria with potential probiotic properties, in the test arm. They also saw a decrease in *Prevotella *and an increase in *Roseburia *in participants with reduced CRP levels. In another study, improvements in gut microbiome diversity and evenness were seen by the significant increase in Shannon diversity in the personalized diet group [13].

Personalized Pharmacological Interventions

Personalized pharmacological interventions based on microbiome profiles for drug metabolism and efficacy: Changes in the gut microbiome can influence how various drugs are metabolized, which may alter their efficacy and toxicity (increase or decrease). Literature reports that the same medication produces different effects because people have unique compositions of gut microbiota. The research on berberine, nonsteroidal anti-inflammatory drugs (NSAIDs), and histamine-2 blockers by Madhogaria et al. (2022) demonstrates how gut microbiota affects drug responsiveness variability. Tungstate treatment, which specifically targets gut *Enterobacteriaceae*, effectively improved colitis, demonstrating how selective microbial interventions can control disease [42].

Trøseid et al. (2020) proposed a “drug the bug” strategy for microbial enzymatic pathway inhibitors that produce harmful metabolites like TMAO, although drugs would not need to kill bacteria specifically. Future drug treatments may be manufactured to address particular microbial behaviors through gut microbiome-based assessment of individual patients. The same source indicates that drug effectiveness should be adjusted according to each person's unique gut microbiome [43].

Valles-Colomer et al. (2023) examined how gut microbiota affects statin efficacy. Multiple studies have established a connection between response to statins and variations found in the human gut microbiome. The gut bacterium *Eggerthella lenta* demonstrates the ability to deactivate the cardiac drug digoxin. The data shows drug therapy can become individualized through microbiome-based assessments to enhance medical benefits while decreasing drug-related side effects [44]. Hu et al. (2021) found that statins improved ACS patients' gut microbiomes and clinical outcomes [27].

FMT and immunotherapy: Madhogaria et al. (2022) mentioned in their study that *Akkermansia muciniphila* correlated with the response of cancer patients to PD-1/PD-L1 blockers [42]. Valles-Colomer et al. (2023) further highlighted that FMT has shown promise in overcoming resistance to anti-PD-1 therapy in melanoma patients, indicating that modulating the gut microbiome through FMT can personalize the response to cancer immunotherapy [44].

Novel therapeutic strategies beyond conventional primary care

Next-generation probiotics and engineered microbes have been helpful in modulating the gut microbiota for health benefits beyond conventional probiotics, with formulations targeting specific molecular pathways and employing synthetic biology.

Advances in Probiotic Formulations Targeting Specific Molecular Pathways

Traditional probiotics mainly include *Lactobacillus *and *Bifidobacterium *species commonly found in fermented foods. However, meta-omics studies are consistently identifying candidates for next-generation probiotics based on more diverse taxa [45]. Next-generation probiotics target particular enzymatic pathways on purpose. *A. muciniphila, Faecalibacterium prausnitzii, Eubacterium hallii, P. copri,* and *Bacteroides *species are interesting next-generation probiotic options for cardiometabolic disease (CMD) [45,46].

Obesity is associated with *A. muciniphila*. Pasteurized *A. muciniphila* postbiotics enhanced insulin sensitivity, insulinemia, and plasma total cholesterol in a pilot experiment. This suggests employing certain microorganisms or their inactivated counterparts for targeted effects, such as reducing obesity through improving insulin sensitivity and reducing plasma cholesterol levels [46]. The butyrate-producing gut bacterium, *F. prausnitzii, *is another next-generation probiotic with anti-inflammatory effects and possible Crohn's disease treatment. Butyrate synthesis activates G protein-coupled receptors (GPCRs) and controls gastrointestinal changes, which may aid obese and diabetic patients. Developing TMA synthesis inhibitors that target certain microbial TMA lyases is one technique to target specific enzymatic pathways. These medications may lower TMAO and reverse atherosclerosis in animal models. Thus, TMA lyase may be a therapeutic target for TMAO regulation. This “drug the bug” strategy targets microbial processes without killing them. Next-generation probiotics are being developed to boost butyrate production, with supplementation options including butyrate-producing bacteria, fiber, or healthy donor feces [46]. *B. longum* subsp. longum modulates *Roseburia* abundance and phosphatidylserine levels, helping to improve intestinal and liver metabolic disturbances induced by high-fat diets. Atherosclerosis and gut microbial LPS synthesis are reduced by *Bacteroides vulgatus* and *dorei*. *Enterococcus faecium* WEFA23 from newborns altered gut microbiota and increased cholesterol 7-alpha-hydroxylase gene expression to reduce high-fat diet-induced hyperlipidemia in rats. By reducing intestinal cholesterol absorption, *L. acidophilus* ATCC 4356 reduced atherosclerosis in apolipoprotein E-knockout mice [46].

Synthetic Biology Approaches to Modulate the Gut Microbiota

The sources introduce the concept of gut microbiome engineering to produce personalized advanced therapeutic medicine. This aligns with synthetic biology approaches. While the term “synthetic biology” is not explicitly detailed in the context of specific examples of engineered microbes in these sources, the general idea of manipulating the microbiome for therapeutic purposes points towards this field [47].

Phage therapy, using bacteriophages to target specific components of the microbiome, is mentioned as a potential approach to modulate specific bacteria without the broad side effects of antibiotics and the risk of antibiotic resistance. This could be considered a form of targeted microbial intervention [48].

The development of “smart probiotics” with functional considerations for novel health and industrial applications suggests an engineering aspect. Research into the genetic sequencing of gut microbiota is expected to change the therapy approach and aid in the reduction of negative events and healthcare costs [49]. In summary, next-generation probiotics and engineered microbes represent a significant advancement in our ability to precisely manipulate the gut microbiota. By focusing on specific molecular pathways and leveraging engineering principles, these approaches hold the potential for more targeted and effective therapeutic interventions for a range of diseases.

FMT and Microbiome Modulation 

FMT and microbiome modulation are being explored for their potential in treating metabolic and CVDs, although the field is still evolving. FMT includes transferring feces from a healthy donor to a recipient to change their gut microbiota. It treats recurrent *Clostridium difficile* infection (CDI). Research is underway for chronic disorders like diabetes, inflammatory bowel disease (IBD), and obesity. In cardiometabolic, FMT is mostly experimental. The impact of FMT from lean donors on insulin sensitivity in obese metabolic syndrome patients may be transient. FMT from vegan donors in metabolic syndrome patients altered gut microbiota but not TMAO production or vascular inflammation [50].

FMT is being investigated beyond CDI. Endoscopic administration limits its usage in acute and high-risk cardiovascular conditions, including ACS and decompensated HF [43]. Some research suggests that FMT can transmit atherosclerosis susceptibility and increase thrombosis potential and platelet reactivity in animal models [51]. Conversely, FMT from wild-type mice to atherosclerosis-prone mice has shown a decrease in atherosclerotic lesions in one study [52].

Adjunct Therapies and Emerging Modalities

Use of bioactive compounds (e.g., polyphenols, prebiotics) in modulating gut flora: Prebiotics and probiotics can cure chronic disorders, including diabetes, IBD, and obesity. These can improve gut microbial profiles and function in certain human investigations. As a prebiotic, a high-fiber diet may reduce intestinal dysbiosis, blood pressure, and cardiac function in animal models of hypertension-induced HF [53,54]. Prebiotics are substrates preferred by host bacteria for health benefits. Probiotics are live strains of chosen microorganisms that improve health when supplied properly. Lactic acid bacteria and yeasts are conventional probiotics. In HF, probiotics may modulate cardiac remodeling and function. In animal models of myocardial ischemia, *L. plantarum* 299v and *L. rhamnosus* GR-1 were cardioprotective [53,54]. The probiotic yeast *Saccharomyces boulardii* decreased systemic inflammation and enhanced left ventricular ejection fraction in chronic HF patients in a short pilot trial [55]. Probiotics strengthen the epithelial barrier, preventing TMAO and LPS24 translocation. LDL and total cholesterol can be lowered by some probiotics. Probiotics have been shown to lower TMAO levels, suggesting they may treat atherosclerosis. Some *Bifidobacterium *strains lower TMAO [56].

Combination therapies integrating microbiome modulation with standard CVD treatments: Drugs targeting the alteration of gut microbiome composition can be a promising therapy for chronic diseases. The efficacy of cardiovascular drugs can be influenced by the gut microbiome, as microbes can metabolize drugs and affect their absorption. Statins, a standard treatment for CVD, have been shown to modulate the gut microbiome and may mediate some of their metabolic and anti-inflammatory effects through these changes [57]. Microbial TMA lyases-targeted TMA inhibitors lower TMAO levels and atherosclerosis in mice, complementing traditional therapies. “Drug the bug” approaches aim to target microbial pathways without being bactericidal, such as inhibiting TMA lyase. Combining dietary changes with microbial or pharmacological interventions may impact the microbiome and serum metabolite levels, offering novel avenues to counter metabolic and cardiovascular disorders. Future cardiovascular risk stratification may include microbiome interventions [43]. Overall, while FMT holds promise for modulating the gut microbiome, its application in metabolic and CVDs requires further research to address safety, standardization, and efficacy. Adjunct therapies like prebiotics and probiotics, as well as targeted interventions affecting microbial metabolism, are also being explored as ways to modulate the gut microbiome in conjunction with standard CVD treatment. A personalized strategy is essential due to the complex interaction between diet, gut microbiome, and host physiology.

Limitations identified in the current literature

There are several limitations to translating microbiota research into clinical practice for metabolic and CVDs despite credible, promising preclinical research data. The limitations are that there are inconsistently defined regimens in interventional studies where intermittent fasting diets were adopted, limiting the comparison of outcomes due to variability. Furthermore, the sample size of current clinical trials is small, restricted to specific geographical regions that limit the generalizability of the findings. Dietary, medication, lifestyle, and comorbidity data are typically missing from gut microbiota investigations, which might skew results. Large inter-individual differences in gut microbiota composition and host metabolic responses to therapies make generalizing results difficult and require tailored methods. While associations between gut microbiota and disease have been identified, establishing direct and causal relationships remains challenging. Animal models are important to establish causality and define mechanisms. Dietary modifications, prebiotics, probiotics, and FMT vary widely. Clinical effectiveness has not always matched animal model results. A major impediment regarding FMT emerges from the lack of standardized procedural guidelines. Standardization and optimization of FMT procedures require proper donor screening methods and delivery protocols. Safety risks from FMT interventions involve the transmission of antibiotic-resistant bacteria. Established screening methods of donors and protocol procedures work to minimize security threats. Medical experts remain uncertain about all unexpected effects that occur when the microbiome changes because of these interventions. Clinical trial monitoring demands strict attention and extended time evaluations for proper evaluation.

Potential directions for future research and clinical applications

Future research holds immense potential for advancing our understanding of the gut microbiome and translating this knowledge into clinical applications for metabolic and CVDs. Conducting large-scale cohort studies with extensive sample sizes and longitudinal data, integrating multiple meta-omics techniques, is crucial to identify robust biomarkers and elucidate the complex interactions within the gut microbiome and its impact on the host. Future research should focus on elucidating the precise molecular mechanisms by which gut microbes and their metabolites influence the pathogenesis and progression of diseases. This includes in vitro and in vivo studies, as well as animal models. Developing personalized dietary recommendations and interventions based on an individual's gut microbiome profile, along with other clinical parameters, holds great promise for disease prevention and treatment. Identifying responders to specific foods and interventions will be key. Future therapeutic strategies will likely focus on more targeted approaches to modulate the gut microbiome, such as next-generation probiotics with specific functions, “drug the bug” approaches targeting specific microbial enzymatic pathways (e.g., TMA lyase), and phage therapy to modulate specific bacterial components. Refining FMT protocols through better donor screening, non-invasive delivery methods (e.g., capsules), and the identification of active microbial consortia will enhance its safety and efficacy. Cultivated microbial mixtures as alternatives to donor stool are also being explored. Meta-omics approaches can be used to identify novel microbial and metabolite biomarkers for early disease diagnosis, risk stratification, prognosis, and prediction of treatment response. Integrative analyses combining different omics layers are particularly promising for biomarker discovery. Investigating the synergistic effects of combining microbiome modulation strategies (e.g., prebiotics, probiotics, FMT, targeted inhibitors) with standard CVD treatments (e.g., statins) may lead to more effective therapeutic interventions. Implementing longitudinal monitoring of the gut microbiome and related metabolites in individuals at risk or with established disease will provide valuable insights into disease progression and the impact of interventions. Continued advancements in sequencing technologies, computational biology, bioinformatics tools, and meta-omics methodologies will be crucial for overcoming current limitations and accelerating progress in the field. Future research needs to further explore the intricate communication pathways between the gut microbiome and distant organs, including the heart, brain, liver, and kidneys, to understand the systemic impact of microbiome alterations. Overall, while significant challenges remain, the rapid advancements in microbiome research and meta-omics technologies offer exciting possibilities for developing novel diagnostic and therapeutic strategies for metabolic and CVDs through targeted modulation of the gut microbiota. A multidisciplinary and collaborative approach involving clinicians, microbiologists, bioinformaticians, and regulatory experts will be essential to realize the full potential of the gut-heart axis in clinical practice.

## Conclusions

The key metabolites are TMAO and SCFAs (butyrate, propionate, and acetate) that influence inflammation, lipid metabolism, and endothelial cell functions. The review found the association of TMAO with atherosclerosis and its potential as a therapeutic target, as well as the anti-inflammatory and lipid metabolism properties of SCFAs. The other microbial metabolites, such as indoxyl sulfate and p-cresyl sulfate, also play a role in causing endothelial dysfunction and atherosclerosis. The complexity between the gut microbiota-heart axis requires meta-omics approaches for analysis because they found variabilities in both CVD-related microbes and metabolites for each individual. This gut-heart axis comprises immune, endocrine, and neuronal pathways in which microbial communities from the gut interact with the heart to affect cardiovascular health. The review highlighted that the complex interplay between gut microbiota composition, host genetics, and epigenetic modifications underscores the need for a personalized medicine approach due to the complicated mechanism of CVD. To devise a personalized approach, integrative multi-omics analysis provides promising findings to identify biomarkers and develop targeted interventions. This integrative multi-omics analysis includes evidence from animal and preclinical studies to develop personalized dietary and pharmacological strategies by making informed microbiome profiles that can optimize cardiovascular outcomes. Furthermore, the implications of FMT, next-generation probiotics, and "drug the bug" strategies are promising and emerging therapeutic approaches.
